# Field Evaluation of Liver Ultrasound Measurements and Biochemical and Metabolic Parameters During the Transition Period in Dairy Cows

**DOI:** 10.3390/ani15142013

**Published:** 2025-07-08

**Authors:** Giorgia Taio, Anastasia Lisuzzo, Silvia Bordin, Matteo Gianesella, Igino Andrighetto, Giorgio Marchesini, Enrico Fiore

**Affiliations:** Department of Animal Medicine, Production and Health (MAPS), University of Padua, Viale dell’Università 16, 35020 Legnaro, Italy; giorgia.taio@phd.unipd.it (G.T.); anastasia.lisuzzo@unipd.it (A.L.); silvia.bordin.vet@gmail.com (S.B.); igino.andrighetto@unipd.it (I.A.); giorgio.marchesini@unipd.it (G.M.)

**Keywords:** transition period, dairy cows, liver, ultrasound, metabolic parameters

## Abstract

Ketosis and fatty liver syndrome are metabolic disorders apparent in dairy cows during the transition period. The study focused on examining how varying levels of milk production in dairy cows might reflect or influence specific blood biochemical markers and liver health as assessed through ultrasonography. A total of 65 Holstein-Friesian cows from six farms were evaluated at three time points as follows: 7 days before expected calving and at 7 and 21 ± 3 days postpartum. Each evaluation included the body condition score (BCS), blood sampling for biochemical analysis, and liver ultrasonography. Based on average farm milk yield, cows were divided into three production groups as follows: GR1 (38.4 ± 6.45 L/day, *n* = 23), GR2 (42.9 ± 2.77 L/day, *n* = 24), and GR3 (45.69 ± 7.49 L/day, *n* = 18). Parameters assessed included liver lipid content and ultrasonographic measurements such as portal vein diameter and depth, liver depth, and liver angle. Significant time-dependent changes were observed in liver size, fat metabolism, and electrolyte balance, especially postpartum. However, no significant differences emerged among the production groups, indicating that these changes likely represent physiological adaptations to lactation. These findings support the use of blood analysis and ultrasonography as practical, minimally invasive tools for routine metabolic health monitoring in dairy cows during the transition period.

## Simple Summary

Dairy cows undergo physiological and metabolic changes during the transition period, which makes them particularly vulnerable to disorders such as ketosis and fatty liver. These conditions can negatively impact both animal welfare and milk production. The aim of this study was to assess whether different milk yields were associated with changes in the biochemical parameters and liver ultrasound findings in dairy cows. This study showed that calving and an increase in days in milk significantly influenced an increase in liver size, fat metabolism, and changes in electrolyte balance, particularly during the postpartum period. These results suggested that the liver undergoes metabolic adjustments to support the energy demands of lactation. However, no significant differences were found between cows from farms with different milk yields, suggesting that these metabolic changes are a general physiological response to lactation rather than being influenced by productivity levels. Furthermore, the study supports the potential use of non-invasive tools like blood tests and ultrasound to monitor liver health and detect early signs of metabolic imbalance in dairy cows.

## 1. Introduction

The transition period in dairy cattle is commonly defined as the period of time from three weeks before calving to three weeks postpartum [1]. Fetal growth already increases energy demands during prepartum while reducing feed intake. Afterwards, colostrum and milk production negatively influence energy balance during dairy cows’ postpartum [2]. Therefore, the cow enters a state of negative energy balance (NEB), which is considered a physiological but potentially harmful adaptation that results in the mobilization of body reserves, particularly adipose tissue [3,4,5]. However, an excessive NEB is considered a negative factor for the animal’s health and productivity due to metabolic diseases and their link with immunosuppression [6].

Lipases in adipose tissue catabolize the triglycerides (TRGs) into glycerol and non-esterified fatty acids (NEFAs), which are transported from the extracellular space to the blood mainly through binding with albumin [7]. The NEFAs captured by the liver can undergo complete oxidation with energy formation, re-esterification into TRGs and export as very low-density lipoprotein (VLDL), or storage in hepatocytes [8]. The complete oxidation of NEFAs generates acetyl-coenzyme A (acetyl-CoA), a metabolite that can be used for energy production through the Krebs cycle. However, the Krebs cycle undergoes an overload in the presence of high amounts of NEFAs to be oxidized as the liver has a limited capacity to metabolize acetyl-CoA [9]. The NEFA cut-off values of 0.29 mEq/L prepartum and 0.57 mEq/L postpartum are commonly used to assess excessive lipomobilization, as values above these thresholds have been associated with an increased risk of metabolic disorders such as hepatic lipidosis and reduced reproductive performance [3,10].

Beyond NEFA overload, nutritional deficiencies in vitamins and glucogenic amino acids can impair the efficiency of the Krebs cycle, even under conditions of para-physiological lipomobilization (<0.29 mEq/L prepartum, <0.57 mEq/L postpartum) [3]. In fact, B-vitamins and glucogenic amino acids serve as essential cofactors and intermediates for the Krebs cycle [11,12]. As a result, the increased levels of acetyl-CoA due to Krebs cycle block are used for the production of the following ketone bodies: acetoacetic acid, acetone, and β-hydroxybutyrate (BHB) [9]. The BHB is the more stable form of ketone bodies found in the blood of dairy cows, and its concentration is an indicator of fatty acid oxidation. On the contrary, NEFA concentrations reflect the level of mobilization of lipid reserves, as compensating for the imbalance between the nutrients the cow obtains from the diet through ingestion and the nutrients that are lost through milk [13]. According to the literature, cows experience a metabolic stress if BHB concentration is equal to or greater than 1.0 mmol/L [3,14].

Thus, the liver plays a central role in the metabolism of dairy cows which are particularly prone to metabolic liver disease [15]. Liver functionality can be assessed through the enzymes’ activity, expressed as the concentration of aspartate aminotransferase (AST), alanine aminotransferase (ALT), γ-glutamyl-transferase (GGT), as well as total bilirubin (T.BIL.) [16,17,18].

Regarding diagnostic imaging, liver ultrasonography is a non-invasive diagnostic technique used in dairy cattle to assess liver health and identify possible diseases, such as lipidosis, hepatomegaly, or liver dysfunction [19]. Previous studies have demonstrated the utility of ultrasonography in estimating hepatic lipid accumulation in dairy cows during the transition period [20,21]. In particular, the integration of B-mode ultrasonography and texture analysis software have shown strong correlations with liver lipid content (TAG) measured via liver biopsy [18,22]. Although liver biopsy remains the gold standard for assessing TAG, it is not a practicable method in farms and is considered invasive, with potential adverse effects [23].

Ultrasonography allows us to visualize the location, size, and appearance of the parenchyma, as well as the topography of major vessels of the organ [19]. Under physiological conditions, the liver ultrasound pattern is homogeneously hypoechogenic and it has an acute angle centered on the caudal vena cava. In addition, the liver has a maximum depth of 15 cm under physiological conditions [24]. Hepatic veins are visible throughout the parenchyma and their diameter increases progressively in the direction of the portal vein and caudal vena cava, while the lumen appears anechoic [25]. The typical appearance of the portal vein is star-shaped upon probe movement due to its branching, and its wall is distinguishable from that of the hepatic veins as it is hyperechogenic. Its position is ventrolateral to the caudal vena cava and its diameter in the last and penultimate intercostal space is between 2.9 and 5.3 cm and tends to decrease in the cranial direction [19]. The vena cava has a position that is usually dorsomedial to the portal vein and has a characteristic triangular shape in section [25].

Some measurements can be made directly on the ultrasound image, with the help of cursors, which can be done quickly and inexpensively in both healthy and sick animals. To do this, the image should be captured at the time of maximum inspiration of the cow [19]. Among the most significant measurements that can be used to assess any liver changes are the depth of the liver (LD) and the depth of the portal vein (DPV) [26]. The LD is defined as the distance between the ventral limit of the internal intercostal and the dorsal limit of the perivisceral adipose tissue, passing through the center of the portal vein. The DPV is defined, instead, as the distance between the ventral margin of the entire intercostal muscle and the center of the vein. Other significant measurements are liver angle and the estimation of the lipid content [26].

The primary objective of this study was to evaluate changes in the biochemical profile and liver ultrasound findings in groups of cows with different levels of productive performance, in order to determine whether productivity influences these parameters. While the metabolic adaptations of transition cows have been widely studied, limited studies have explored the use of liver ultrasonographic measurements as non-invasive indicators of metabolic status, particularly in relation to different production levels. Considering this context, the hypothesis of this study was that cows belonging to production-level groups would display different patterns in both metabolic and ultrasonographic parameters throughout the transition period. Another purpose of this research was to assess if liver ultrasonography could approximate an evaluation of metabolic health status and detect lipomobilization in practical farm conditions. The final aim was to clarify the practical applicability and limitations of liver ultrasound in field settings, which is essential to improve on-farm metabolic health monitoring and early disease detection.

## 2. Materials and Methods

### 2.1. Animals, Farms, and the Study Design

This study was approved by the Animal Welfare Board of the University of Padua (ethical approved protocol number 81/2022). The experimental protocol was reviewed and approved in accordance with the standards recommended by the Guide for the Care and Use of Laboratory Animals and Directive 2010/63/EU. In addition, the farmers were previously informed and agreed with the objectives of the study and the methods used.

The experiment was conducted from March until May 2022 across six Italian Holstein dairy farms located in the Veneto region, specifically in the province of Vicenza. During this period, environmental temperatures remained within the thermal comfort zone for dairy cows, with no recorded heat waves or prolonged high humidity [27]. Each farm was equipped with mechanical ventilation and cow cooling devices, which were regularly operated as part of routine herd management.

All farms had a loose housing system and they adopted a total mixed ration (TMR) feeding strategy formulated to meet the nutritional requirements of dairy cows during both the dry and early lactation periods [28]. Although formulations were adjusted based on each farm’s forage availability and production targets, the overall composition was comparable among farms. The base of the ration consisted of maize silage and alfalfa hay or silage. Additional ingredients included cereal silages (e.g., ryegrass, wheat, or barley), grass hay, straw, maize (in meal form or as high-moisture grain silage), protein supplements such as soybean or sunflower meal, and mineral–vitamin premixes. The average composition of the total mixed ration (TMR) provided to cows was as follows (expressed in g/kg of dry matter): dry matter (DM), 497.7; crude protein (CP), 148.7; neutral detergent fiber (NDF), 332.3; acid detergent lignin (ADL), 31.7; and starch, 258.3. The average digestibility of the ration nutrients was as follows: DM, 0.592; CP, 0.570; NDF, 0.329; and starch, 0.979.

All animals received the TMR twice daily and had continuous access to fresh water. Cows in different physiological stages—prepartum and postpartum—were provided with phase-specific TMR formulations tailored to their distinct nutritional requirements. For the lactating cows, the average diet composition in all the farms follows the recent nutrient recommendations for early and mid-lactating Holstein cows [29] for crude protein (150–170 g/kg DM), NDF (280–320 g/kg DM), and starch (202–260 g/kg DM). The consistency of this feeding approach across farms was previously monitored in a related study involving the same facilities, which included periodic assessments of TMR composition and management practices [30]. Cows were dried 55 ± 5 days before expected calving.

All animals in dry periods and within two weeks of scheduled calving were clinically examined before each procedure. Their health status was assessed by measuring the rectal temperature, heart rate, and respiratory rate and assessing the appetite and fecal consistency. Animals with clinical signs of metabolic disease such as ketosis, hypocalcemia, fatty liver disease, displaced abomasum, and rumen acidosis were excluded from the trial. Of these healthy animals, 65 dairy cows were randomly selected from the six different farms.

Specifically, 12 animals were enrolled from farm A (4 primiparous, 8 pluriparous), 12 from farm B (5 primiparous, 7 pluriparous), only primiparous from farm C (n = 6), 17 from farm D (8 primiparous, 9 pluriparous), 7 from farm E (1 primiparous, 6 pluriparous), and 11 from farm F (2 primiparous, 9 pluriparous) for a total of 20 primiparous and 45 pluriparous. All cows in the transition period from each farm were enrolled consecutively based on their availability during the study period. A stratified sampling strategy to equalize the number of primiparous and multiparous cows across farms was not applied. Therefore, the distribution of parity reflects the natural composition of each herd at the time of data collection.

The study design applied in this study was longitudinal. All enrolled animals were evaluated at the following three time points during the transition period: 7 ± 3 days prepartum (T0) considering the expected date of calving; 7 ± 3 postpartum (T1); and the third and final evaluation was carried out at 21 ± 3 postpartum (T2). The T0 sampling captures the phase of reduced dry matter intake and increasing metabolic demand. The first week postpartum represents the peak of metabolic stress, with the highest risk of negative energy balance and associated disorders. The third week postpartum corresponds to the early lactation stage, when cows begin to metabolically stabilize and adapt to lactation demands. This time frame is widely adopted in transition cow studies and aligns with previous research focused on metabolic and inflammatory dynamics in this period [1,4,13,31].

All animals were assessed for BCS (1–5 scale; [32]) and underwent blood sampling, and trans-abdominal ultrasound examination of the liver at each time point (T0, T1, and T2).

The BCS evaluation was performed by the same trained veterinarian at all time points. The evaluator was not blinded to the farm of origin; however, since the classification into productivity groups was performed retrospectively based on milk yield data, the scorer was unaware of group allocation during data collection.

All samplings were performed in the morning, approximately 4 to 6 h after the routine morning feeding and milking, to minimize short-term post-prandial effects and ensure comparable physiological conditions across farms. Moreover, production data were retrospectively collected.

The average productive performance of each farm at T2 (21 ± 3 postpartum) was 40.3 ± 3.67 L/day (farm A), 44.7 ± 9.64 L/day (farm B), 46.7 ± 5.35 L/day (farm C), 43.8 ± 8.13 L/day (farm D), 42.1 ± 12.05 L/day (farm E), and 36.6 ± 9.23 L/day (farm F).

Afterwards, farms were ranked by milk production and divided into three groups (tertiles), each containing two farms. The low production group (GR1) included farm A and farm F with a mean production of 38.4 ± 6.45 L/day (range 20–52 L/day; *n* = 23); the group with intermediate values (GR2) included farm D and farm E with a mean production of 42.9 ± 2.77 L/day (range 28–68 L/day; *n* = 24) and high the production group (GR3) included farm B and farm C with a mean production of 45.69 ± 7.49 L/day (range 30–60 L/day; *n* = 18).

### 2.2. Blood Sampling and Biochemical Analysis

Blood samples were obtained from the coccygeal vein using an evacuated tube system and stored in Venosafe tubes (9 mL; Terumo Venosafe, Leuvel, Belgium) containing a clot activator. Immediately after collection, tubes were kept refrigerated at 4 °C and transported within one hour to the laboratory of the Department of Animal Medicine, Production and Health of the University of Padua in Legnaro, using a portable freezer (CoolFreeze CFX65 W professional, Dometic, Stockholm, Sweden) maintained at approximately −22 °C. Upon arrival, samples were centrifuged at 3000 rpm (1750 g-force, Heraeus Labofouge 400, Thermo Scientific, Milan, Italy) for 10 min at room temperature (~20°C) to obtain serum. Serum was aliquoted into 1.5 mL Eppendorf tubes (four aliquots per animal and time point) and stored at −20°C until analysis. Freeze–thaw cycles were strictly avoided by using aliquots to prevent repeated thawing of the same sample. All procedures followed a standardized protocol to ensure sample integrity and minimize pre-analytical variability.

A complete biochemical analysis of serum was performed for each animal and each time point (T0, T1, and T2) by automatic clinical chemistry analyzer (BT3500; Biotecnica Instruments S.p.a., Rome, Italy). NEFA concentration was determined by a colorimetric method, NEFA RX Monza test (kit no. FA 115; Randox, Crumlin, UK); BHB concentration was determined by RANBUT RX Monza test (kit no. RB 1007; Randox, Crumlin, UK). Moreover, serum samples were also analyzed for the quantification of AST (IU/L), total bilirubin (T.BIL.; mg/dL), NEFAs (mEq/L), BHB (mmol/L), total protein (TP; g/L), urea (mg/dL), glucose (GLU; mg/dL), cholesterol (CHO; mg/dL), alanine aminotransferase (ALT; IU/L), gamma glutamyl transferase (GGT; IU/L), alkaline phosphatase (ALP; U/L), creatine (CRE; mg/dL), phosphorus (P; mg/dL), calcium (Ca; mg/dL), magnesium (Mg; mg/dL), chlorine (Cl; mmol/L), creatine kinase (CPK; IU/L), lactic-dehydrogenase (LDH; IU/L), albumin (ALB; g/L), potassium (K; mEq/L), and sodium (NA; mEq/L).

### 2.3. Liver Ultrasonographic Evaluation

The ultrasound examination of the liver was performed on the right side of the animal at the level of the 10th intercostal space using the MylabOneVET portable ultrasound scanner (ESAOTE S.p.A., Genoa, Italy) equipped with a multifrequency convex probe (Animal Science Probe, SC3421 2.5–6.6 MHz; ESAOTE S.p.A., Genoa, Italy).

For each ultrasound scan, the same settings were maintained for the liver scan as follows: 2.5 MHz frequency, 26 cm depth, maximum gain, and neutral time-gain position [25]. The area selected for ultrasonographic examination, on the right side of the animal between the intercostal spaces, was cleaned and wetted with 90% ethyl alcohol as a transducer agent. The probe was then moved dorsoventrally about 15 cm into the intercostal space. The liver was examined for focal lesions, including abscesses, neoplastic masses, or abnormal lipid infiltrates. Multiple ultrasound images were archived in medical uncompressed (DICOM) format for further analysis.

Survey of LD (mm), PVD (mm), DPV (mm), and liver angle on caudal vena cava (°) [26] were measured and calculated for each animal and in each time point using MyLab^TM^ Desk^3^ software package (ESAOTE S.p.A., Genoa, Italy). In addition, texture assessment of the liver parenchyma was performed using MaZda analysis software version 4.6 (Technical University of Lodz, Institute of Electronics, Lodz, Poland) to estimate the TAG (mg/g) using the formula already compared to the gold standard of liver biopsy and developed on the MylabOneVET handheld scanner [25]. All the ultrasonographic examinations were performed by the same experienced veterinarian using a standardized scanning protocol to ensure consistency across all animals and time points. To assess repeatability, each measurement was taken twice during the same session, and the average value was used for statistical analysis.

The analysis was performed according to the software developer’s instructions (co-occurrence matrix: 6 bits/pixel; gradient features: 8 bits/pixel; run length matrix: 4 bits/pixel; and wavelet transform: 12 bits/pixel). To minimize the effects of both over-brightness and image contrast variations on the texture analysis result, a normalization of the gray level histogram was calculated with MaZda by fitting the histogram data into μ ± 3σ (μ: gray level mean; σ: gray level standard deviation) [25].

### 2.4. Statistical Analysis

The “rcmdr” package of R software version 4.4.2 [33] was used for statistical analysis. The distribution of the data was evaluated using the Shapiro–Wilk normality test and the Levene variance test. All analyzed variables met the assumptions of normality and homogeneity of variance, as assessed by the Shapiro–Wilk and Levene’s tests, respectively. For this reason, statistical analysis was carried out according to a two-way ANOVA with a mixed model that used group, parity, time, and their interaction as fixed factors; animal effect was used as random and repeated factors. In this model, the term “milk-group” refers to clusters of farms categorized based on their average milk production levels (low, medium, high), while parity and time refer to primiparous vs. multiparous cows and sampling time points, respectively.

A post hoc pairwise comparison of the least squares means was performed using Bonferroni correction. Additionally, Spearman’s rank correlation analysis was performed to assess the relationship between the considered variables. Correlation coefficients were interpreted as follows: |r| ≥ 0.70 were considered strong correlations, 0.30 ≤ |r| < 0.70 moderate correlations, and |r| < 0.30 weak correlations [34]. A *p*-value of ≤0.05 was considered statistically significant, and *p*-values 0.05 < *p* ≤ 0.10 were considered indicative of a trend to significance. The prevalence of ketosis and lipomobilization among the production groups (GR1, GR2, and GR3) was analyzed using a chi-squared (χ^2^) test in MedCalc Statistical Software (version 23.2.0). In order to assess the statistical power of the between-group comparisons at each individual time point, a post hoc power analysis was conducted with the G*Power software (version 3.1.9.7).

Furthermore, the predictive ability of biochemical and ultrasonographic parameters for lipomobilization status was evaluated using logistic regression models. Each parameter was fitted as the independent variable and a binary outcome representing lipomobilization as the dependent variable. The binary variable was defined based on NEFA thresholds [3] (NEFA > 0.29 mmol/L prepartum and NEFA > 0.57 mmol/L postpartum). Additionally, the same analysis was performed using BHB thresholds (BHB > 0.60 mmol/L prepartum and BHB > 1.20 mmol/L postpartum) to define ketosis.

The “goodness-of-fit” of each model was assessed using the Hosmer–Lemeshow test with 10 groups. The test allowed the evaluation of how well each parameter alone predicted the lipomobilization status.

## 3. Results

The effect of parity (primiparous vs. multiparous) did not result in statistically significant differences for any of the variables considered (*p* > 0.10). Therefore, results are presented without distinction between parity groups.

All the variables are expressed in the following tables as the mean and standard error of the mean (SEM), respectively. The BCS values and biochemical parameters are shown in Table 1, Table 2 and Table 3. A significant time effect was evidenced for seventeen parameters (*p* < 0.001 for BCS, NEFAs, BHB, T.BIL., AST, GLU, CHO, TRGs, ALT, GGT, ALP, CRE, LDH, Mg, Cl, K, and Na). It is important to highlight that no significant differences were found between the productivity groups (GR1, GR2, and GR3) for the majority of biochemical and ultrasonographic parameters analyzed. Indeed, a significant group effect was shown only with ALT (*p* < 0.001). The post hoc power analysis confirmed sufficient power for detecting differences in milk production between groups (power = 0.968).

The BCS was initially high, with an average of 3.50 across all three groups at T0. It subsequently decreased as the animals progressed through the study period to a range of 3.00–3.25 at T2. The NEFA and BHB levels were low in the prepartum period, peaked at T1, and then declined to 0.36 ± 0.1 and 0.80 ± 0.06 mEq/L at T2, respectively. No significant differences between production-level groups were observed for these metabolites. However, a significant group effect was found for ketosis prevalence at T2 (*p* < 0.05), with the medium-production group (GR2) showing a notable decrease in prevalence from 25.0% at T1 to 4.17% at T2.

An interaction effect approaching significance was observed for T.BIL levels (*p* = 0.080). While all groups showed an increase at T1, the extent of this increase varied as follows: GR1 exhibited the highest peak at 0.29 ± 0.03 mg/dL, whereas GR2 and GR3 showed more moderate increases, reaching 0.20 mg/dL and 0.22 mg/dL, respectively. The AST levels increased at T1 compared to the lowest values observed at T0. Although they decreased at T2, they remained higher than in the prepartum period. Regarding GLU, the highest concentrations were observed in the prepartum period at T0 (60.6 mg/dL). The CHO levels, on the other hand, reached their peak at T2. Notably, differences between T0 and T1 were found only in GR3. Regarding TRGs, the highest values were observed at T0 (mean = 21 mg/dL for the three groups). In contrast, GGT showed a consistent increase across the study period, peaking at T2. ALP, however, followed a downward trend, starting at its highest level at T0 and reaching its lowest value at T2. Considering the renal parameters, CRE values were higher at T0 in all groups, followed by a decrease at T2. While GR2 showed a reduction already at T1, GR1 and GR3 maintained similar values between T0 and T1 before decreasing at T2. The LDH concentrations were highest at T2 for all groups. GR1 showed a progressive increase from T0 to T2, with no significant differences observed between the time points (*p* = 0.643). In GR2 and GR3, LDH values increased steadily across the study period, with significant differences observed between T0 and T1 (*p* < 0.001). An interaction effect was not detected (*p* = 0.737). Concerning mineral profile, Mg exhibited a fluctuating pattern, showing similar concentrations at T0 and T2, with a drop in the immediate postpartum period. Cl had higher concentrations in the prepartum phase, followed by a decrease after calving, with no differences between T1 and T2. Both GR1 and GR2 had a decrease in K level from prepartum to postpartum period on the contrary of GR3 which K concentration remained stable over the study. Despite the change over time, no differences were found among groups at 7 days before calving and 7 days after calving. Instead, both GR2 and GR3 had a greater level of K at 21 days postpartum compared to GR1. In contrast, Na showed a continuous decline across the three time points, with progressively lower values from T0 to T2.

ALT values were highest in GR1 at T0, followed by a progressive reduction at T1 and T2. In contrast, GR2 and GR3 showed more stable values over time. Additionally, an interaction effect (*p* < 0.001) was detected. Specifically, GR1 showed the highest ALT values at T0, followed by a progressive decrease at T1 and T2. In contrast, both GR2 and GR3 maintained more stable ALT levels across all time points, with only minimal variations.

Instead, a significant interaction between group and time was discovered for five parameters (Urea, *p* = 0.019; CHO, *p* = 0.015; ALT, *p* < 0.001; P, *p* < 0.001; K, *p* < 0.001), and two parameters (BIT, *p* = 0.08; Na, *p* = 0.054) displayed a trend to significance for interaction.

The liver ultrasound parameters are shown in Table 4. The PVD (*p* = 0.007) and LD (*p* < 0.001) had a significant time effect, instead TAG showed a trend to significance (*p* = 0.094) for the interaction between group and time. GR1 showed a marked increase in LD at T1 and GR2 demonstrated a similar pattern. GR3 showed a smaller increase. Despite significant temporal changes, no significant group effect was observed.

Results of the chi-square test comparing the prevalence of ketosis and postpartum lipomobilization at 7 and 21 days in milk across different groups are shown in Table 5 and Table 6.

**Figure 1:**
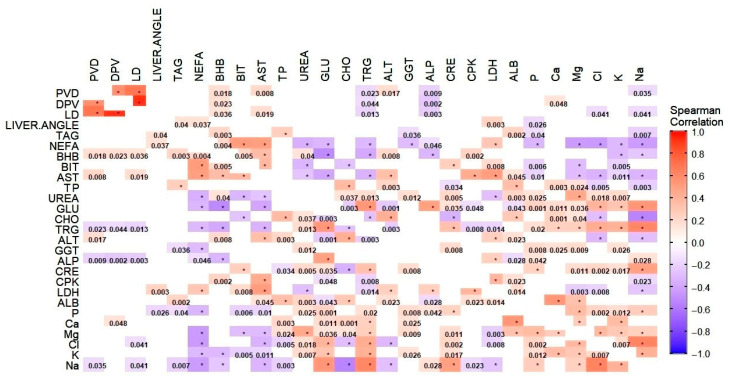
Results of the Spearman rank correlation analysis. Only correlations with a *p*-value ≤ 0.05 were considered statistically significant and included as colored square in the plot. Shades of red indicate a positive correlation approaching 1, while shades of blue represent a negative correlation approaching −1. Significant *p*-values are shown within the squares. * *p*-value < 0.001. ALB (albumin), ALP (alkaline phosphatase), ALT (alanine aminotransferase), AST (aspartate aminotransferase), BHB (β-hydroxybutyrate), BIT (total bilirubin), CHO (cholesterol), CPK (creatine kinase), CRE (creatine), DPV (portal vein diameter), GGT (γ-glutamyl-transferase), GLU (glucose), LD (liver depth), LDH (lactic dehydrogenase), NEFAs (non-esterified fatty acids), TP (total protein), PVD (portal vein depth), TAG (total liver lipid), TRGs (triglycerides).

**Figure 2:**
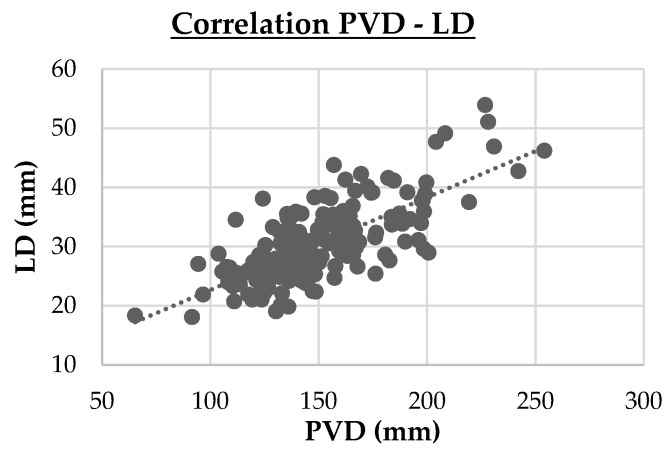
Cluster map of the strong Spearman correlation (|r|≥0.70 ) between portal vein diameter (PVD) and liver depth (LD). PVD vs. LD (r = 0.74, *p* < 0.001)

**Figure 3:**
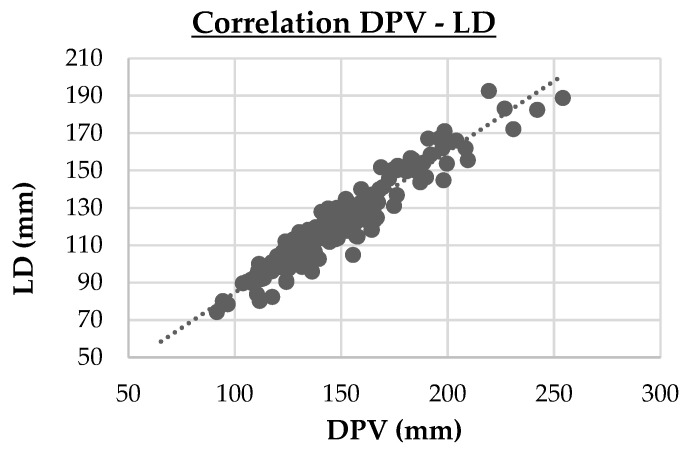
Cluster map of the strong Spearman correlation (|r|≥0.70 ) between the depth of portal vein (DPV) and liver depth (LD). DPV vs. LD (r = 0.95, p < 0.001)

**Figure 4:**
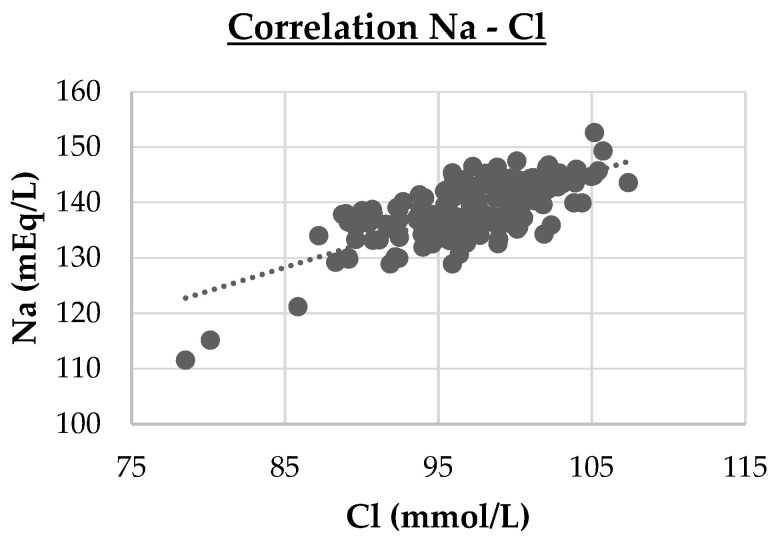
Cluster map of the strong Spearman correlation between sodium (Na) and chloride (Cl). Cl vs. Na (r = 0.714, *p* < 0.001).

The heat map in Figure 1 shows the result of Spearman correlations analysis. Correlation coefficients were calculated across the full dataset, regardless of group assignment. Only correlations with a *p*-value ≤ 0.05 were considered statistically significant and are displayed as colored squares. Shades of red indicate positive correlations (approaching +1), while shades of blue represent negative correlations (approaching −1). The color intensity reflects the strength of the correlation. Statistically significant *p*-values are shown within the squares. Correlation coefficients were interpreted as follows: |r| ≥ 0.70 were considered strong correlations, 0.30 ≤ |r| < 0.70 moderate correlations, and |r| < 0.30 weak correlations [34]. The analysis revealed the following:

- Three strong correlations (PVD vs. LD, r = 0.74, *p* < 0.001; DPV vs. LD, r = 0.95, *p* < 0.001; Cl vs. Na, r = 0.714, *p* < 0.001). These are highlighted in cluster maps shown in Figure 2, Figure 3 and Figure 4, which allow for a clearer visualization of the progression and consistency of these associations between the parameters.
- Forty-six moderate correlations, with five of these tending to the moderate (AST vs. LDH, r = 0.641, *p* < 0.001; PVD vs. DPV, r = 0.633, *p* < 0.001; AST vs. CPK, r = 0.587, *p* < 0.001; GLU vs. Na, r = 0.584, *p* < 0.001; ALB vs. Ca, r = 0.583, *p* < 0.001; TRGs vs. Na, r = 0.566, *p* < 0.001).
- A total of 109 weak correlations (|r| < 0.30).

Overall, the strongest correlations were observed among ultrasonographic parameters (LD, DPV, and PVD), as well as between some biochemical parameters involved in hepatic and electrolyte balance (Cl and Na).

The results of Hosmer–Lemeshow test are showed in Table 7. The logistic regression models based on NEFA thresholds showed acceptable goodness of fit for BHB (χ^2^ = 10.2, *p* = 0.025), LD (χ^2^ = 25.75, *p* = 0.001), TRGs (χ^2^ = 18.33, *p* = 0.019), and total bilirubin (T.BIT) (χ^2^ = 19.63, *p* = 0.012). On the contrary, the models involving the remaining parameters do not demonstrate significant *p*-values (>0.05).

When using BHB-based thresholds, acceptable goodness of fit was observed for ALP (χ^2^ = 20.95, *p* = 0.007), CHO (χ^2^ = 13.84, *p* =0.086), TRGs (χ^2^ = 24.86, *p* = 0.001), and NEFAs (χ^2^ =30.47, *p* <0.001).

## 4. Discussion

In recent decades, it has been shown that the NEB represents a para-physiological adaptation of the animal to the high energy demands imposed by calving and subsequent lactation [26]. However, poor adaptation of the cow to this period may promote the onset of metabolic diseases. Metabolic stress negatively impacts animal welfare, manifesting as decreased feed intake, increased disease susceptibility, and altered behavior [35]. For this reason, the assessment of key biochemical parameters of the transition period represents a minimally invasive method established for investigating the response of cows and diagnosing diseases, in particular ketosis and hepatic lipidosis [3].

The main purpose of this study was to assess these changes across groups with different productivity levels to determine whether productivity influences these variations. Additionally, it aimed to evaluate biochemical and liver ultrasound changes in cows during the transition period. By incorporating routine biochemical and ultrasonographic assessments into herd health programs, veterinarians and farmers can identify metabolic imbalances earlier, potentially reducing the incidence and severity of metabolic diseases. Early detection and management not only support productivity but also contribute to improved animal welfare by minimizing discomfort, preventing disease progression, and promoting faster recovery.

### 4.1. BCS and Energy Metabolism

Considering the BCS of the animals, all groups started with an average of 3.50 (5-point scale) in the prepartum period and ended with a value of 3.00–3.25 three weeks after calving. The pattern observed over time aligns with the physiological loss of body reserves. [36]. The optimal body condition of dry cows should be between scores 3.00 and 3.75 (5-point scale). Risk of ketosis can be reduced when cows have scores from 3.25 to 3.50 (5-point scale) at calving. In early lactation, cows lose BCS but should not lose more than 1.00 unit [37]. In this study, no differences were detected between groups, in contrast to previous studies. The BCS at calving and subsequent BCS reduction were previously associated with milk production [38]. Specifically, greater BCS at calving was negatively associated with milk yield, as well as a greater BCS reduction during early lactation [39]. In this study, no differences in BCS were detected between groups suggesting that milk yield was not influenced by the BCS at calving and its reduction. However, the absence of such associations in our dataset may be explained by the relatively homogeneous BCS values across farms and the limited sample size, which might have reduced our ability to detect subtle differences between groups.

Regarding biochemical parameters, NEFA and BHB levels were higher in the postpartum period compared to the pre-calving phase. These temporal patterns reflected the mobilization of fatty acids and production of ketone bodies in response to the increased energy demands of lactation [13]. In our study, the prevalence of ketosis one week after calving was similar across groups with different milk production levels.

However, a significant reduction in ketosis prevalence was observed at three weeks postpartum in the medium-production group (GR2), which showed a significant reduction in ketosis prevalence (from 25.0% at T1 to 4.17% at T2, *p* < 0.05). This reduction may reflect a more effective restoration of metabolic homeostasis following an initial period of negative energy balance and lipid mobilization. This pattern suggests that medium-producing animals may present physiological mechanisms that enable a more efficient adaptation to the metabolic demands of early lactation. This metabolic profile appears more favorable not only when compared to high-producing cows, which may experience prolonged metabolic stress, but also relative to low-producing cows. Notably, average BHB concentrations did not differ significantly between groups at 21 days after calving (GR1: 0.86 ± 0.1 mEq/L; GR2: 0.61 ± 0.1 mEq/L; GR3: 0.93 ± 0.11 mEq/L; *p* > 0.10), despite the change in ketosis prevalence, suggesting a broadly comparable metabolic adaptation immediately after calving [40]. However, these findings remain speculative and require further investigation to elucidate the underlying biological mechanisms associated with different levels of milk production.

Blood glucose dynamics show notable changes throughout the transition period [41]. Based on our data, glucose concentrations remained within the physiological range (50–80 mg/dL) [42] during the prepartum but dropped slightly below the lower limit postpartum. Immediately after calving, glycemia drops due to the sudden rise in glucose demand for lactose synthesis in milk. The observed decrease in glucose concentrations at 7 days post-calving associated with an increase in the levels of NEFAs and BHB represents the increase in energy metabolism due to the NEB. If animals persistently experience a hypoglycemic state (<40 mg/dL), the risk of clinical and subclinical ketosis increase [43]. In our study, no animals showed hypoglycemia immediately after calving. However, no differences were observed between groups in excessive lipomobilization (17–39%) or ketosis (25–39%) prevalence at 7 days postpartum. Consequently, glucose concentration should not be interpreted independently, but it must be assessed in association with BHB and NEFA levels to gain a more comprehensive understanding of the overall metabolic condition also in relation to milk yield.

### 4.2. Liver Parameters

Liver function is evaluated by the blood concentration of bilirubin, AST, GGT, and ALT observed at the plasma level [44]. As for BIL.T., the physiological range is between 0.1 and 0.5 mg/dL [42]. In the study, all groups did not exhibit BIL.T. concentrations exceeding the pathological cut-off of 1 mg/dL. The mean concentrations of AST and GGT were lower prepartum and increased significantly in the immediate postpartum period in the present study. The highest AST activity was recorded one week after calving (T1), while the greatest level of GGT was evidenced at three weeks after calving (T2). This result is consistent with the findings reported by Stojević et al. (2005) [45]. The cut-off commonly used to discriminate the possible presence of liver damage is AST > 100 U/L [46]. The average AST concentrations were within the physiological range at any time. Consequently, the increase in AST level immediately after calving was due to an increased liver activity due to lipomobilization without important liver damage in all groups. However, average GGT levels were above the upper limit of the reference range during postpartum (6.1–17.1 U/L) [42]. This slight increase in GGT may be associated with mild hepatic stress or temporary dietary disturbances that do not severely affect liver function. Furthermore, increases in serum AST and GGT were seen to be associated with a linear increase in milk yield by Giannuzzi et al. [47].

Even though all ALT means levels fell within the physiological range (11–40 U/L) [42], a significant difference was observed in the group with low milk yield. Animals with lower production (GR1) exhibited higher values one week before calving, followed by a gradual decrease in the early postpartum period. This observation aligns with Tainturier et al. (1984), who reported that ALT activity decreased from the dry period to early lactation [48]. However, the difference based on productivity disagreed with the findings of Jóźwik et al. (2012), who reported that ALT levels were lower in animals with a lower milk yield [49]. In cattle, ALT is not generally considered a specific or reliable marker of hepatocellular injury, due to its low basal hepatic activity and the significant contribution of extrahepatic sources such as muscle and cardiac tissue [42]. Although the study identified differences among groups with different levels of milk production, its interpretation should be approached with caution, as ALT is not a liver-specific enzyme in cattle. The observed changes may instead reflect general metabolic or muscular adaptation rather than hepatocellular recovery.

The reference range of serum ALP activity in cattle is very broad and has been shown to be 0–488 U/L [42]. This value was determined without regard to lactation. It is probable that the broad range of reference values for cattle is due to the high level of serum ALP activity in lactating cows [50]. In our data, ALP levels remained within the physiological range, but a significant increase (*p* < 0.001) was observed seven days before calving (slightly above the cut-off [42]), followed by a decrease during the postpartum period. The increase during the final stages of pregnancy are due to increased osteoblastic activity as the animal prepares for milk production and bone mineralization [51]. However, it is of little value in hepatic disease because of the broad range of reference values with which the patient’s values must be compared [42]. The lack of differences in ALP levels in relation to milk yield (*p* = 0.464) could be explained by the multifactorial nature of its regulation, which is not necessarily linked to hepatic function or milk production. As previously noted, ALP has a limited diagnostic value for liver function in cattle, as its regulation is multifactorial and influenced by various physiological, hormonal, and metabolic factors that extend beyond hepatic health or milk production itself [50].

### 4.3. Lipidic Metabolism

Focusing on the lipid profile, including CHO and TRGs, significant changes occurred during the transition period in the study. The CHO is used for the synthesis of lipoproteins, which facilitate fatty acid transport, as well as the production of steroid hormones essential for energy metabolism regulation [52]. In fact, Kim and Suh [53] reported that an energy deficit during the first month after calving, as determined by severe BCS loss, was associated with lower CHO concentrations, suggesting that serum CHO may be a useful predictor of energy balance status during early lactation. In this study, a progressive increase in CHO level was evidenced in all groups from one week before calving to the following three weeks. The combination of high CHO levels and low prevalence of lipomobilization at three weeks after calving (T2) suggests that the animals are not actively mobilizing fat stores. This condition could indicate a positive sign of metabolic recovery after calving with an early restoration of energy balance regardless of milk production levels.

Moving on to TRGs (physiological range is 0–14 mg/dL) [42], their levels were above the normal range immediately before calving followed by a normal condition during postpartum. Other studies confirm that serum TRGs are highest in the late dry period with a significant fall postpartum, reach their lowest concentrations in early lactation, with no differences among lactating groups [54]. The drastic energy demand for the onset of lactation causes NEFA levels to rise, while plasma TRG levels remain low or experience a slight reduction. This is due to the liver’s limited capacity to export TRGs in cows in the form of very-low-density lipoproteins (VLDL) [55], which may predispose the animal to fat accumulation in the liver and the development of hepatic steatosis [56]. The low TRG levels observed in early lactation could be the result of TRGs being broken down into NEFAs to meet the increased energy demands associated with milk production [57]. Thus, the absence of differences between groups could be due to the overall metabolic processes being sufficiently robust to handle the increased energy needs, and these processes may not vary significantly between animals with different levels of milk production.

### 4.4. Renal Parameters

In general, CRE levels in cattle are usually between 1.0 and 2.0 mg/dL [42]. In this study, the average CRE concentrations did not exceed the upper limit of the range, and a decreasing pattern was observed from the prepartum to postpartum. Indeed, a decrease may be observed after calving linked to the intensifying mobilization of energy substrates and amino acids for muscle [58]. At time points T1 and T2, both CRE concentrations and BCS were at their lowest levels. This concomitant reduction suggests that the lower CRE values may be attributed to a decrease in muscle mass reflecting a catabolic state associated with NEB in the postpartum period [59]. Since no significant differences in BCS were observed between the groups with different milk yields, the lack of differences in CRE concentrations across groups appears consistent. This parallel pattern supports the hypothesis that CRE levels are more closely related to body tissue reserves, particularly muscle mass, rather than to milk production.

Notably, LDH levels were found to be above the physiological cut-off (692–1445 U/L) [42]. In the days leading up to calving, an increase in LDH levels can be observed, which may reflect a higher cellular turnover. Findings suggested that the total activity of LDH was higher in late lactating cows followed by early lactating and late pregnant cows [60]. Considering the comparable BCS and similar lipomobilization across the groups, the lack of variation in LDH concentrations with respect to milk production supports the fact that LDH is more reflective of systemic postpartum adjustments than of lactation intensity.

### 4.5. Mineral Profile

Details on the chemical and physical composition of the Total Mixed Ration (TMR) used in the farms recruited for the present study can be found in Andrighetto et al. (2023) [30]. In relation to minerals, no values were found to exceed the established reference ranges (P 5.6–6.5 mg/dL; Mg 1.8–2.3 mg/dL; Cl 97–110 mEq/L; K 3.9–5.8 mEq/L; Na 132–152 mEq/L) [42]. In our data, the time effect on *p* levels was only observed in the high-production group. At T1 (one-week postpartum), a significant interaction was observed, with high-producing cows (GR3) showing lower serum *p* concentrations compared to lower-producing groups. This finding may reflect the higher metabolic demand for phosphorus required for milk synthesis during early lactation [61], particularly in high-yielding animals. Given that the diet composition was similar across all groups, this temporal variation is driven by the physiological requirements of intense milk production, rather than by changes in intake.

Serum Mg concentrations were found to be reduced at T1 (one-week postpartum) across all groups, regardless of milk yield. As Mg status is primarily influenced by dietary intake and ruminal absorption [62], and all cows were fed a comparable diet, the observed decline is likely due to a transient postpartum reduction in dry matter intake or absorption efficiency, rather than a differential demand based on productivity.

Cl, K, and Na were higher in pre-calving and then decrease after calving. In fact, K levels may fluctuate during the transition period but generally tend to decrease in the postpartum phase due to increased mobilization and excretion through urine. Additionally, K loss through saliva and feces may be accentuated during metabolic stress and dietary changes [63]. The Na levels typically remain stable during the transition period but can be influenced by changes in water balance, as well as dietary intake. It plays a significant role in regulating blood Na levels, as sodium is essential for maintaining fluid balance and proper nerve and muscle function [64]. The pattern of mineral concentration changes over time was similar among the different groups, indicating that milk yield did not significantly influence the dynamics of mineral metabolism in the transition period.

Therefore, it can be observed that the biochemical analysis of the main metabolic parameters is a suitable tool for assessing the adaptation of dairy cattle to the NEB during the transition period in this study. Nevertheless, the results confirm physiological processes of the transition period, already present in the literature, but without differences between milk yield.

### 4.6. Liver Ultrasound Measurements

Following the biochemical analysis, the focus was shifted to the ultrasound measurements of the liver. Both the estimated lipid content and ultrasound measurements serve as useful indicators for assessing liver health and identifying potential pathological conditions, primarily hepatic lipidosis [26]. However, the diagnostic value of liver measurements for lipidosis can often be limited due to the high individual variability among cows [65].

Both PVD and DPV are ultrasonographic measurements used as indirect indicators of liver volume and perfusion. Variations in these parameters may reflect hepatic congestion, lipid infiltration, or altered hemodynamic, all of which are relevant during the transition period when the liver undergoes metabolic adaptation [65,66]. The results of this study regarding PVD showed an increase in this value from prepartum to the first postpartum in low and high production groups and they agree with a previous study [24]. The mean PVD values did not deviate excessively from the lower limit of the physiological range 2.9–5.3 cm indicated by Braun (2009) [19]. According to Giannuzzi et al. (2021), variations in PVD and metabolites related to liver function, such as AST and BIL.T., are promising indicators of liver metabolic changes, and as such, should be further investigated for lipidosis diagnosis [26]. In all the groups of this study, the mean PVD value did not deviate significantly from the lower limit of the physiological range of 29–53 mm, as indicated by Braun (2009) [19]. The lack of differences in productivity levels observed in our study has also been reported in previous research, where no relevant associations were observed between the US parameters and milk production traits, including when expressed in terms of productivity [66].

Regarding LD, the groups with medium and high productivity did not show differences over the course of the experiment. In both postpartum time points, the low and high productivity groups of our study maintained average values above the cut-off of 152.6 mm [24]. It is suspected that the greater LD is associated with significant lipomobilization in the weeks immediately following parturition, as indicated by Mahdi Komeilian et al. (2011) [67]. However, owing to the anatomical positioning of the portal vein, its measurements serve as indicators of liver size, which, in animals in a healthy physiological state, is to some extent correlated with their overall body size [66].

In the present study, across all groups and assessment time points, the estimated TAG mean value was predominantly below the range indicative of moderate lipidosis (50–100 mg/g), except for one group at a single time point, where the value was slightly above the over cut-off [20,22]. No significant effects were observed over time or between groups, but an interaction was noted. This interaction may indicate a more complex relationship between TAG and other parameters, such as BHB or NEFAs. Indeed, the synthesis and accumulation of TAG in the liver are related to the concentration of NEFAs in the blood [40].

These findings are in line with previous studies that suggest that changes in liver size and blood flow occur as part of the metabolic adjustments needed to support the increased nutrient demands of lactation [24]. Therefore, changes in PVD, DPV, liver angle, and LD values were of little use in distinguishing between low- and high-producing animals, although some of these parameters showed a time effect. Indeed, a key limitation of this study is the lack of significant group effects in many of the biochemical markers, which may suggest that individual variability within groups or other uncontrolled factors could have influenced the results.

Despite the potential of ultrasonography as a non-invasive tool for liver investigation, certain limitations must be acknowledged. One of the main concerns is the inherent operator dependency of ultrasound examination, which may affect image quality, measurement consistency, and interpretation [68]. Factors such as probe positioning and pressure applied to the transducer can influence the accuracy of measurements, especially when performed under field conditions where cow posture, movement, or environmental constraints may reduce repeatability [69]. Furthermore, differences in rumen fill, fat deposition, or anatomical variation between animals may also introduce measurement variability, particularly for parameters such as portal vein diameter or liver size [66].

To minimize these effects, all ultrasound examinations in this study were performed by a single experienced operator using a standardized scanning protocol and probe placement. Nevertheless, some variability is unavoidable, and future studies should consider incorporating inter- and intra-observer variability analyses to better quantify the reproducibility of liver ultrasound measurements in cattle.

Although this field-based study presents some inherent methodological constraints, such as the grouping of cows according to farm-average milk yield and the relatively small sample sizes per farm, these aspects reflect the practical realities of on-farm research. While the absence of a formal random effect for ‘farm’ may limit the statistical generalizability of inter-group comparisons, our approach allowed for the exploration of metabolic and ultrasonographic patterns relevant to different production contexts. Future studies should consider larger sample sizes and more controlled experimental conditions, including standardized diets and environments, to minimize confounding factors.

Furthermore, few biochemical and one ultrasonographic parameter showed potential as indicators of lipomobilization, the variability in the regression model fit suggests that no single marker can fully capture the metabolic status. Our findings were consistent across both NEFA- and BHB-based classifications of lipomobilization and ketosis, although different parameters showed predictive ability depending on the definition used. Specifically, BHB, LD, TRGs, and total bilirubin were significant when using NEFA thresholds, while ALP, cholesterol, TRGs, and NEFAs emerged as significant under BHB thresholds. This supports the notion that a combination of biomarkers, possibly integrated with imaging data, is necessary to improve diagnostic accuracy. Moreover, the lack of strong predictive power for the other variables may be influenced by individual animal variation, farm management practices, and the dynamic physiological changes occurring during transition. These factors emphasize the need for further research to validate and refine reliable, non-invasive tools for early metabolic health monitoring on farms. Additionally, stratified analyses considering genetic backgrounds and parity could provide deeper insights into the metabolic adaptations and ultrasonographic variations observed in dairy cows during the transition period.

## 5. Conclusions

In conclusion, this study offers comprehensive insights into the dynamic metabolic adaptations that occur during the transition period in dairy cows. However, given the minimal group-level differences and the study’s methodological constraints—including the grouping approach and sample size—these findings should be considered preliminary and exploratory.

While overall differences between groups were minimal, significant temporal changes were observed in a broad range of biochemical parameters, including liver enzymes, lipid metabolites, and electrolyte profiles, highlighting the complex physiological shifts during this critical period. While these results support the potential utility of ultrasonographic monitoring in on-farm metabolic assessments, the limited variations observed across groups highlight the need for further investigation to fully elucidate the biological significance and practical implications of these changes. In the study, ultrasonographic parameters such as PVD and LD were assessed as potential indicators of liver metabolic status. However, no significant correlations were found between these ultrasonographic measures and the biochemical markers of lipomobilization, as NEFAs and BHB. This lack of correlation suggests that ultrasonography and biochemical analyses may provide complementary information on different facets of hepatic adaptation during the transition period. Further research is necessary to clarify the relationship between these diagnostic tools and to establish their combined utility in the metabolic monitoring of dairy cows.

The consistent metabolic trends across different productivity groups suggest that these adaptations represent a generalized physiological response to the metabolic demands of the transition period and early lactation, rather than being directly influenced by milk yield levels. Although some parameters showed group-time interactions, overall differences between farms categorized by production level were minimal. This finding underscores the multifactorial nature of metabolic status in transition cows, where individual variability likely plays a significant role beyond milk production alone—such as genetic background or farm-level management practices.

To sum up, the hypothesis that different production levels would be associated with distinct biochemical and liver ultrasonographic changes during the transition period was not confirmed. The absence of significant differences between productivity groups indicates that other factors beyond milk yield may play a more critical role in the metabolic status of transition dairy cows.

## Figures and Tables

**Table 1:** Results from linear mixed model (*p*-values) for the body condition score (BCS). Results from the linear mixed model (*p*-values) and least square means for non-esterified fatty acids (NEFAs), beta-hydroxybutyrate (BHB), glucose (GLU), total bilirubin (T.BIL.), aspartate aminotransferase (AST), alanine aminotransferase (ALT), gamma-glutamyl transferase (GGT), alkaline phosphatase (ALP), cholesterol (CHO) and triglycerides (TRGs). The first group (GR1; 38.4 ± 6.45 L/day) represented the farms with the lowest average productivity, the second (GR2; 42.9 ± 2.77 L/day) included those with intermediate values, and the third (GR3; 45.69 ± 7.49 L/day) comprised the farms with the highest productivity levels.

Parameter	Group	T0 (−7 ± 3 Days)	T1 (+7 ± 3 Days)	T2 (+21 ± 3 Days)	SEM ^1^	*p*-Values		
						Group	Time	Interaction
BCS ^2^ (1/5)	GR1	3.50 (3.50–4.00; 3.00–4.25)	3.25 (3.00–3.69; 2.75–4.50)	3.00 (2.75–3.50; 2.75–4.00)	/	0.550	<0.001	0.566
	GR2	3.50 (3.00–3.75; 2.25–4.50)	3.00 (2.75–3.62; 2.75–4.25)	3.00 (2.75–3.25; 2.50–4.00)	/			
	GR3	3.50 (3.31–3.75; 3.00–4.00)	3.25 (3.06–3.50; 2.75–3.75)	3.25 (3.00–3.25; 2.75–3.75)	/			
NEFAs (mEq/L)	GR1	0.18 ^y^	0.45 ^x^	0.30 ^y^	0.06	0.725	<0.001	0.785
	GR2	0.24 ^y^	0.49 ^x^	0.37 ^xy^	0.06			
	GR3	0.23 ^y^	0.55 ^x^	0.43 ^x^	0.06			
BHB (mmol/L)	GR1	0.64 ^y^	0.98 ^x^	0.86 ^x^	0.10	0.778	<0.001	0.273
	GR2	0.54 ^y^	0.79 ^x^	0.61 ^y^	0.10			
	GR3	0.59 ^y^	1.07 ^x^	0.93 ^x^	0.11			
GLU (mg/dL)	GR1	60.5 ^x^	47.0 ^y^	46.1 ^y^	1.72	0.994	<0.001	0.100
	GR2	60.6 ^x^	52.8 ^y^	51.6 ^y^	1.85			
	GR3	60.7 ^x^	45.6 ^y^	47.9 ^y^	1.99			
T.BIL. (mg/dl)	GR1	0.18 ^y^	0.29 ^a,x^	0.15 ^y^	0.03	0.791	<0.001	0.080
	GR2	0.16	0.20 ^b^	0.16	0.03			
	GR3	0.17	0.22 ^b^	0.17	0.05			
AST (U/L)	GR1	50.5 ^y^	77.4 ^x^	61.5 ^y^	6.06	0.991	<0.001	0.897
	GR2	51.5 ^y^	75.5 ^x^	63.2 ^xy^	6.23			
	GR3	50.7 ^y^	80.8 ^x^	68.2 ^x^	6.47			
ALT (U/L)	GR1	22.2 ^a,x^	18.1 ^a,y^	17.2 ^y^	1.06	<0.001	<0.001	<0.001
	GR2	15.3 ^b^	15.5 ^b^	17.1	1.14			
	GR3	14.6 ^b^	15.2 ^b^	16.8	1.22			
GGT (U/L)	GR1	19.2 ^y^	21.6 ^y^	24.6 ^x^	1.45	0.579	<0.001	0.553
	GR2	17.3 ^y^	20.5 ^x^	20.6 ^x^	1.60			
	GR3	19.2 ^y^	21.5 ^xy^	24.2 ^x^	1.62			
ALP (U/L)	GR1	146 ^x^	124 ^xy^	110 ^y^	11.1	0.220	<0.001	0.464
	GR2	162 ^x^	149 ^x^	120 ^y^	12.3			
	GR3	134 ^x^	117 ^xy^	107 ^y^	12.6			
CHO (mg/dL)	GR1	88.0 ^y^	87.0 ^y^	140 ^b,x^	4.78	0.281	<0.001	0.015
	GR2	79.4 ^y^	79.8 ^y^	149 ^b,x^	5.11			
	GR3	77.9 ^z^	91.1 ^y^	160 ^a,x^	5.55			
TRGs (mg/dL)	GR1	20.8 ^x^	9.40 ^y^	9.12 ^y^	1.28	0.408	<0.001	0.360
	GR2	22.3 ^x^	9.88 ^y^	10.7 ^y^	1.31			
	GR3	19.8 ^x^	10.5 ^y^	10.8 ^y^	1.36			

Values are expressed as the mean ±SEM (^1^ Standard Error of the Mean). ^2^ BCS (Body condition score) is presented as median (first to third quartile; minimum to maximum). Letters ^a–b^ within the same column indicate statistically significant differences between groups at the same time point (*p* ≤ 0.05). Letters ^x–z^ within the same row indicate statistically significant differences over time within the same group (*p* ≤ 0.05).

**Table 2:** Results from a linear mixed model (*p*-values) and least square means for creatinine (CRE), urea, creatine phosphokinase (CPK), lactate dehydrogenase (LDH), total protein (TP), and albumin (ALB). The first group (GR1; 38.4 ± 6.45 L/day) represented the farms with the lowest average productivity, the second (GR2; 42.9 ± 2.77 L/day) included those with intermediate values, and the third (GR3; 45.69 ± 7.49 L/day) comprised the farms with the highest productivity levels.

Parameter	Group	T0 (−7 ± 3 Days)	T1 (+7 ± 3 Days)	T2 (+21 ± 3 Days)	SEM ^1^	*p*-Values		
						Group	Time	Interaction
**CRE** **(mg/dL)**	GR1	1.28 ^x^	1.24 ^x^	1.06 ^y^	0.04	0.820	<0.001	0.109
	GR2	1.31 ^x^	1.14 ^y^	1.06 ^y^	0.04			
	GR3	1.29 ^x^	1.22 ^x^	1.07 ^y^	0.05			
**Urea (mg/dL)**	GR1	23.9	21.8	24.9	2.06	0.261	0.112	0.019
	GR2	27.9 ^x^	18.9 ^y^	25.7 ^x^	2.10			
	GR3	23.6	21.1	26.1	2.17			
**CPK** **(U/L)**	GR1	113	195	158	33.8	0.440	0.227	0.847
	GR2	133	148	169	35.1			
	GR3	176	234	195	38.4			
**LDH** **(U/L)**	GR1	1441	1720	1762	164	0.643	<0.001	0.737
	GR2	1583 ^y^	1959 ^x^	1844 ^xy^	177			
	GR3	1354 ^y^	1658 ^xy^	1713 ^x^	177			
**TP** **(g/L)**	GR1	64.9	64.7	67.3	1.39	0.931	0.210	0.821
	GR2	64.6	66.4	69.7	1.48			
	GR3	65.3	66.0	69.0	1.58			
**ALB** **(g/L)**	GR1	31.3	31.7	31.5	0.53	0.591	0.770	0.405
	GR2	31.1	31.2	32.5	0.56			
	GR3	31.9	32.7	33.3	0.60			

Values are expressed as the mean ± SEM (^1^ Standard Error of the Mean). Letters ^a–b^ within the same column indicate statistically significant differences between groups at the same time point (*p* ≤ 0.05). Letters ^x–y^ within the same row indicate statistically significant differences over time within the same group (*p* ≤ 0.05).

**Table 3:** Results from the linear mixed model (*p*-values) and least square means for minerals: phosphorus (*p*), calcium (Ca), magnesium (Mg), chloride (Cl), potassium (K), and sodium (Na). The first group (GR1; 38.4 ± 6.45 L/day) represented the farms with the lowest average productivity, the second (GR2; 42.9 ± 2.77 L/day) included those with intermediate values, and the third (GR3; 45.69 ± 7.49 L/day) comprised the farms with the highest productivity levels.

Parameter	Group	T0 (−7 ± 3 Days)	T1 (+7 ± 3 Days)	T2 (+21 ± 3 Days)	SEM ^1^	*p*-Values		
						Group	Time	Interaction
**P** **(mg/dL)**	GR1	6.04	5.74 ^a^	5.51	0.39	0.299	0.175	<0.001
	GR2	5.90	5.37 ^ab^	5.96	0.40			
	GR3	6.73 ^x^	4.78 ^b,y^	5.57 ^y^	0.41			
**Ca** **(mg/dL)**	GR1	9.03	8.70	8.88	0.15	0.601	0.163	0.169
	GR2	9.05	8.56	8.80	0.16			
	GR3	8.84	9.00	9.02	0.17			
**Mg** **(mg/dL)**	GR1	2.67 ^x^	2.35 ^y^	2.56 ^x^	0.06	0.510	<0.001	0.751
	GR2	2.62 ^x^	2.40 ^y^	2.58 ^x^	0.06			
	GR3	2.57 ^x^	2.34 ^y^	2.54 ^x^	0.08			
**Cl** **(mmol/L)**	GR1	100 ^x^	95.4 ^y^	95.7 ^y^	0.59	0.766	<0.001	0.539
	GR2	100 ^x^	95.1 ^y^	94.9 ^y^	0.87			
	GR3	100 ^x^	95.0 ^y^	95.5 ^y^	0.94			
**K** **(mEq/L)**	GR1	4.65 ^x^	4.30 ^y^	4.12 ^b,y^	0.08	0.479	<0.001	<0.001
	GR2	4.58 ^x^	4.32 ^y^	4.33 ^a,y^	0.08			
	GR3	4.50	4.31	4.44 ^a^	0.09			
**Na** **(mEq/L)**	GR1	143 ^x^	139 ^y^	131 ^z^	1.04	0.672	<0.001	0.054
	GR2	144 ^x^	137 ^y^	133 ^z^	1.07			
	GR3	144 ^x^	138 ^y^	134 ^z^	1.13			

Values are expressed as the mean ± SEM (^1^ Standard Error of the Mean). Letters ^a–b^ within the same column indicate statistically significant differences between groups at the same time point (*p* ≤ 0.05). Letters ^x–y^ within the same row indicate statistically significant differences over time within the same group (*p* ≤ 0.05).

**Table 4:** Results from linear mixed model (*p*-values) and least square means for portal vein diameter (PVD), portal vein depth (DPV), liver depth (LD), liver angle, and predicted liver triacylglycerol (TAG). The first group (GR1; 38.4 ± 6.45 L/day) represented the farms with the lowest average productivity, the second (GR2; 42.9 ± 2.77 L/day) included those with intermediate values, and the third (GR3; 45.69 ± 7.49 L/day) comprised the farms with the highest productivity levels.

Parameter	Group	T0 (−7 ± 3 Days)	T1 (+7 ± 3 Days)	T2 (+21 ± 3 Days)	SEM ^1^	*p*-Values		
						Group	Time	Interaction
**PVD** **(mm)**	GR1	29.4 ^y^	32.4 ^xy^	34.2 ^x^	1.87	0.834	0.007	0.281
	GR2	28.4	31.8	29.4	1.92			
	GR3	27.8 ^y^	30.5 ^xy^	31.5 ^x^	2.05			
**DPV** **(mm)**	GR1	123	128	127	5.11	0.327	0.470	0.980
	GR2	114	119	116	5.46			
	GR3	123	131	127	5.84			
**LD** **(mm)**	GR1	138 ^y^	159 ^x^	160 ^x^	7.65	0.625	<0.001	0.573
	GR2	138	150	145	7.96			
	GR3	147	161	154	8.54			
**Liver** **angle (°)**	GR1	76.3	75.8	77.3	2.23	0.884	0.857	0.923
	GR2	77.8	80.2	80.0	2.28			
	GR3	77.5	78.7	80.9	2.52			
**TAG (mg/g)**	GR1	73.2	80.7 ^b^	83.1	7.95	0.950	0.310	0.094
	GR2	73.5 ^y^	72.6 ^b,y^	92.4 ^x^	8.07			
	GR3	71.1 ^y^	101.1 ^a,x^	86.4 ^xy^	8.94			

Values are expressed as the mean ± SEM (^1^ Standard Error of the Mean). Letters ^a–b^ within the same column indicate statistically significant differences between groups at the same time point (*p* ≤ 0.05). Letters ^x–y^ within the same row indicate statistically significant differences over time within the same group (*p* ≤ 0.05).

**Table 5:** Results of the chi-square test comparing the prevalence of ketosis (blood BHB ≥ 1.0 mmol/L) at 7 and 21 days in milk across different groups. The first group (GR1; 38.4 ± 6.45 L/day) represented the farms with the lowest average productivity, the second (GR2; 42.9 ± 2.77 L/day) included those with intermediate values, and the third (GR3; 45.69 ± 7.49 L/day) comprised the farms with the highest productivity levels.

Ketosis		
Group	T1 (+7 ± 3 Days)	T2 (+21 ± 3 Days)
GR1	34.8% (n = 23)	21.7% ^a^ (n = 14)
GR2	25.0% ^x^ (n = 17)	4.17% ^b,y^ (n =3 )
GR3	38.9% (n = 26)	33.3% ^a^ (n = 22)

Letters ^a–b^ within the same column indicate statistically significant differences between groups at the same time point (*p* ≤ 0.05). Letters ^x–y^ within the same row indicate statistically significant differences over time within the same group (*p* ≤ 0.05).

**Table 6:** Results of the chi-square test comparing the prevalence of ketosis postpartum lipomobilization (blood NEFAs ≥ 0.57 mEq/L) at 7 and 21 days in milk across different groups. The first group (GR1; 38.4 ± 6.45 L/day) represented the farms with the lowest average productivity, the second (GR2; 42.9 ± 2.77 L/day) included those with intermediate values, and the third (GR3; 45.69 ± 7.49 L/day) comprised the farms with the highest productivity levels.

Lipomobilization		
Group	T1 (+7 ± 3 Days)	T2 (+21 ± 3 Days)
GR1	17.4% (n = 12)	4.35% (n = 3)
GR2	25.0% (n = 17)	8.33% (n = 5)
GR3	38.9% (n = 26)	16.7% (n = 11)

**Table 7:** Hosmer–Lemeshow goodness-of-fit test results for logistic regression models evaluating the ability of selected biochemical parameters to predict lipomobilization or ketosis status in transition dairy cows. The binary outcomes were defined using two different classification criteria: lipomobilization based on NEFA thresholds (NEFAs > 0.29 mmol/L prepartum and > 0.57 mmol/L postpartum) and ketosis based on BHB thresholds (BHB > 0.60 mmol/L prepartum and >1.20 mmol/L postpartum). Only parameters with *p*-values < 0.10 are reported. A *p*-value > 0.05 indicates acceptable model fit. Degrees of freedom for all tests = 8. The significant results included albumin (ALB), β-hydroxybutyrate (BHB), calcium (Ca), lactate dehydrogenase (LD), total bilirubin (T.BIL), triglycerides (TRGs), alkaline phosphatase (ALP), cholesterol (CHO), and non-esterified fatty acids (NEFAs).

Lipomobilization Based on NEFAs			
Parameter	Chi-Squared (χ^2^)	Degrees of Freedom	*p*-Value
BHB	10.2	8	0.025
T.BIL	19.63	8	0.011
TRGs	18.32	8	0.018
ALB	13.65	8	0.091
Ca	14.31	8	0.073
LD	25.74	8	0.001
Ketosis based on BHB			
Parameter	Chi-Squared (χ^2^)	Degrees of Freedom	*p*-Value
NEFAs	30.47	8	<0.001
CHO	13.84	8	0.086
ALP	20.95	8	0.007
TRGs	24.86	8	0.001

## Data Availability

The data presented in this study are available upon request from the corresponding author. The data are not publicly available since they are still under analysis for further publications.
